# Potential of probiotics in controlling cardiovascular diseases

**DOI:** 10.4103/0975-3583.74267

**Published:** 2010

**Authors:** Rajiv Saini, Santosh Saini, Sugandha Sharma

**Affiliations:** *Department of Periodontology and Oral Implantology, Rural Dental College, Loni, Maharashtra, India*; 1 *Department of Microbiology, Rural Dental College, Loni, Maharashtra, India*; 2 *Department of Prosthodontics, Rural Dental College, Loni, Maharashtra, India*

Sir,

The World Health Organization and the Food and Agriculture Organization of the United Nations have defined probiotics as “live microorganisms, which, when administered in adequate amounts, confer a health benefit on the host.” They are also called “friendly bacteria” or “good bacteria.” The concept of probiotics arose at the turn of the 20th century from a hypothesis first proposed by the Noble Prize-winning Russian scientist, Elie Metchnikoff. Most probiotics fall into the group of organisms known as lactic acid-producing bacteria, and are normally consumed in the form of yogurt, fermented milks or other fermented foods.[1,2] The valuable effects of food with added live microbes (probiotics) on human health and, in particular, of milk products, are being increasingly promoted by health professionals, and these probiotics can play an important role in systemic health of the person.

Coronary heart disease (CHD) is caused by a narrowing of the coronary arteries that feed the heart. It is the most common form of heart disease, affecting some 7 million Americans, and it is also the number-one killer of both men and women. Each year, more than 500,000 Americans die of heart attacks caused by CHD. Many of these deaths could be prevented because CHD is related to certain aspects of lifestyle. Some of the risk factors for CHD, or things that increase your risk of developing the disease, are high blood pressure, high blood cholesterol, smoking, obesity, physical inactivity, diabetes and stress. Experimental data are convincing in support of the hypothesis that prebiotics inhibit hepatic lipogenesis in rats and, consequently, induce a significant hypotriglyceridemic effect. Hypothesized mechanisms of this effect include the metabolic effects of short-chain carboxylic acids and the lowering of glycemia or insulinemia,[3] because a metabolic link was recently shown between insulin resistance and the associated risk factors for atherosclerotic cardiovascular disease, especially hypertriglyceridemia, and because of the growing awareness that hypertriglyceridemia itself may be a risk factor in atherogenesis. The potential functional effects described, primarily of prebiotics, need to be studied carefully in humans, especially in conditions known to be associated with hyperinsulinemia and hypertriglyceridemia.[3]

Probiotics support the development of regulatory T cells in the immune system. Dendritic cells (DC) are antigen-presenting cells found throughout the gut that continually sample enteric antigens and present them to naive T cells, resulting in T-cell activation and differentiation. DC is able to discriminate between different microbial strains through the expression of pattern-recognition receptors that recognize specific pathogen-associated molecular patterns. Microbes will stimulate DC to acquire signals to drive the development of either T_H_1, T_H_2 or T_reg_ cell responses. Probiotic bacteria induce a pattern of maturation of DC characterized by the release of small amounts of tumor necrotic factor-α and IL-12, with increased levels of IL-10, and inhibit the generation of proinflammatory T_H_1 cells. Bifidobacteria in particular induce an upregulation of IL-10 production by DC and decrease the expression of the costimulatory molecules, CD80 and CD40. This increase in IL-10 production may act both by having direct antiinflammatory effects and by enhancing the generation of T_reg_ cells. In contrast to the effects of Bifidobacteria, some Lactobacilli strains tend to generate a phenotype of DC characterized by increased costimulatory marker expression but low production of proinflammatory cytokines.[4] These T cells help reduce overzealous immune responses throughout the body. What that can mean are fewer number of white blood cells being sent to a damaged artery and, therefore, less inflammation and less chance of accumulation of cholesterol in the area. Probiotics help reduce blood cholesterol in three different ways:

Probiotics create acids that counter cholesterol production: As probiotic bacteria absorb fiber from the intestines, they generate acids. One of the specific acids, i.e. proprionic acid, reduces production of cholesterol by the liver.

Probiotics break down liver bile acids: Bile acids assist body in digesting fats, and the liver produces these bile acids from cholesterol. The liver recycles bile acids and utilizes them over and over. Probiotics break down bile acids and, therefore, the liver has to make additional bile acids, using up more cholesterol in the progression.

Probiotics actually eat cholesterol: Probiotic bacteria have been shown to break down cholesterol and use it for nourishment.

Thus, it can be concluded that probiotics can be included in foodstuffs through new technologies like microencapsulation and immobilized cell technologies. Further research in this area may offer exciting avenues in health care strategies.[2] Scientific evidence exists to indicate that there is potential for the derivation of health benefits from consuming food containing probiotics. However, it was felt that additional research data are needed to confirm a number of these health benefits in humans. this bacterium in the mouth and other parts of the human body? Indian J Dent Res 2009;20:129.
